# Optimization of *Metarhizium koreanum* MN031-Mt 46: Nutritional Supplementation to Improve Conidia and Cuticle-Degrading Enzyme Production by Solid-State Fermentation

**DOI:** 10.4014/jmb.2412.12079

**Published:** 2025-04-27

**Authors:** Suleiman Abba Muazu, Payorm Cobelli, Teerada Wangsomboondee

**Affiliations:** 1 Center of Excellence in Environment and Plant Physiology, Department of Botany, Faculty of Science, Chulalongkorn University, Bangkok 10330, Thailand; 2 Program in Botany, Faculty of Science, Chulalongkorn University, Bangkok 10330, Thailand; 3 Rice Research and Development Division, Rice Department, Ministry of Agriculture and Cooperatives, Bangkok, 10900, Thailand; 4 Department of Botany, Faculty of Science, Taraba State University, Jalingo 660213, Nigeria

**Keywords:** *Metarhizium koreanum*, solid-state fermentation, performance indices, cuticle-degrading enzyme, response surface methodology

## Abstract

This study aimed to evaluate five different mixed agricultural wastes as potential substrates for solid-state fermentation (SSF) to produce conidia of *Metarhizium koreanum* MN031-Mt 46. Single-factor experiments and a Box-Behnken design (BBD) were employed to optimize the fermentation conditions for enhanced conidia yield. Results indicated that a mixed substrate comprising broken rice and rice bran significantly enhanced the optimal production of aerial conidia of MN031-Mt 46. Optimal fermentation conditions established through response surface methodology (RSM) revealed that with the addition of shrimp shell waste to the mixed substrate, conidia production increased to 8.45 × 10^8^ conidia per gram of dry substrate at 26.19°C temperature, 39.76% moisture, and 1.45% of shrimp shell waste after 301.87 h of incubation. Enhanced conidia performance indices were observed, including higher conidia weight, increased water content, and reduced residue post-harvest. The optimized fermentation conditions resulted in enhanced cuticle-degrading enzymatic activities, with maximum activities of 58.78 ± 2.29 U g^-1^ ds for protease, 126.57 ± 6.47 U g^-1^ ds for lipase, and 58.32 ± 0.78 U g^-1^ ds for chitinase. These findings highlight the potential and versatility of mixed SSF using cost-effective agricultural waste for biopesticide and hydrolytic enzyme production, while promoting sustainable waste management and environmental pollution control, aligning with circular economy principles.

## Introduction

The application of entomopathogenic fungi (EPF) in pest management is an environmentally sustainable practice that serves as an alternative to synthetic pesticides. Synthetic pesticides have harmful effects on the environment, non-target organisms, and human health [1, 2]. In contrast, biopesticides comprising biochemicals, semiochemicals, microbes, enzymes, plant extracts, and other natural substances are recognized for their minimal impact on human health and the environment [3]. Their usage has increased significantly due to restrictions on chemical pesticides, policy goals for organic farming, and farmer’s recognition of their benefits [4-6]. *Metarhizium* species, a type of EPF, are known to infect various host insects by adhering to and penetrating the insect cuticle, ultimately disrupting the host's immune system [7, 8]. Despite their potential as biological control agents, challenges in the scalability and efficiency of mass-producing EPF like *Metarhizium* species persist. Therefore, optimizing conidia and cuticle-degrading enzyme production is crucial for the effective use of these biocontrol agents.

A key prerequisite for developing a pathogen as a biopesticide is its suitability for mass production [9, 10]. *Metarhizium* species can be mass-produced on various substrates. The fungal conidia can be produced as blastospores in submerged fermentation (SmF) or aerial conidia in solid-state fermentation (SSF). SSF is commonly used due to its cost-effectiveness and simplicity in producing aerial conidia [11]. Utilizing agricultural waste as a substrate for conidia and enzyme production reduces costs and provides both a nutrient source and physical support for fungal growth [12]. Thailand, as an agricultural country, has an abundance of starch-rich agricultural and crustacean waste that can be utilized for the conidia production of EPF. Canton [13] reported an annual production of approximately 9.3 billion tons of crustaceans, including shrimp, prawns, and lobsters. Southeast Asia alone produced approximately 1.5 million tons of these [14], including Thailand. Based on consumption demand, 45-48% of shrimp are discarded as waste, which includes the shell (body carapace) and head [15]. Crustacean shell wastes are frequently discarded in landfills or in the sea [16]. The importance of developing sustainable methods to add value to these abundant and inexpensive renewable resources cannot be overstated. Therefore, agricultural and seafood waste utilization for conidia and cell-wall degrading enzyme production would be economically viable, despite its role in controlling environmental pollution and solving the problem of waste disposal [17].

The production of suitable biological control products depends on efficient microbial growth and enzymatic production which is influenced by fermentative conditions [18]. Several factors influence the mass production of biocontrol agents in SSF, including temperature, pH, inoculum concentration, and initial moisture content. Variations in these abiotic factors affect the growth, sporulation, and germination of fungal conidia. To maximize conidia production, the optimization of these culture conditions is imperative. In this regard, Response Surface Methodology (RSM), a multivariate mathematical model, has been extensively applied for optimizing multiple production variables to predict the optimal condition necessary for SSF processes. It provides an analysis of the interaction between the response of interest and several variables with a small number of experiments [19, 20]. Optimization of the variables in a fermentation process can give information about the main effects of the variables and the interaction between variables at varying levels [21]. This optimization is crucial for maximizing conidia production, efficiency, and overall process performance. Understanding these dynamics can lead to more effective fermentation strategies and improved outcomes in biopesticide production.

EPF such as *Beauveria bassiana* and *M. anisopliae* have been reported to produce a wide range of extracellular enzymes that disrupt the primary barrier of the insect cuticle, which is composed of proteins, chitin, and lipids [22]. As the insect cuticle is the initial barrier encountered by EPF, these fungi secrete extracellular enzymes to break down the main components of the cuticle [23-25]. Proteases, chitinases, and lipases, which are cuticle-degrading enzymes, facilitate EPF penetration through the insect cuticle [26] and are considered virulence factors [27, 28]. The production of these enzymes is interrelated [29, 30] and can be induced on the insect cuticle under abiotic conditions [31-33]. Various studies have demonstrated that nutrient supplementation significantly impacts conidia growth, tolerance, sporulation, pest control, extracellular enzyme production and biodegradation [21, 34]. There is a notable gap in the literature regarding the holistic effects of shrimp shell waste or molasses supplementation in *M. koreanum* substrates on conidia production, performance metrics, and enzymatic activity. This study aims to address this gap by optimizing the conidia production of mutagenized *M. koreanum* MN031-Mt 46 using the Box-Behnken design (BBD) of RSM. Additionally, it evaluates the performance metrics and production of cuticle-degrading enzymes (chitinase, lipase, and protease) of the optimized fermented conidia through SSF.

## Materials and Methods

### Fungal Isolates and Inoculum Preparation

Fungal isolates used in this study have been reported by Muazu *et al*. [35]. This study utilized the thermotolerant *M. koreanum* mutant (MN031-Mt 46) and its corresponding wild-type (Wt: MNMHN031) isolates. The mutant and the Wt were recovered from brown plant hopper cadavers on Sabouraud dextrose yeast agar (SDAY) + dodine (n-Dodecylguanidine acetate) (DOD) medium. The SDAY + DOD medium was prepared according to the previously described method of Hernández-Domínguez *et al*. [36] with some modifications.

A 10 mm plug from SDAY + DOD medium was transferred to a fresh SDAY medium without DOD and incubated for 7 days at 16: 8 h photoperiod. Ten milliliters of 0.05% Tween 80 were added into each petri plate and conidia was harvested by gently scraping the surface of the plate with a sterile loop before dispersing into 50 ml falcon tubes. Concentration of the conidial suspension was determined using the Neubauer chamber (Brand 717810) and adjusted to 10^6^ conidia/ml. The conidial suspension 10 ml was added to 90 ml of Sabouraud dextrose broth (SDB) medium in 250 Erlenmeyer flasks and the flasks were incubated in an orbital shaker at 150 rpm at 28±1°C for 96 h. The suspension was used as inoculum for SSF.

### Raw Materials

Five different solid substrates (banana pseudostem, corncob, water hyacinth, sugarcane bagasse, and white broken rice) mixed with rice bran in a ratio of 8: 2 (w/w) along with supplements (shrimp shell waste and molasses) were evaluated for conidia production. Substrates such as white broken rice, rice bran, corncob, and water hyacinth were obtained from local markets, while banana pseudostem and sugarcane bagasse were sourced from locally grown areas in Bangkok, Thailand. Banana pseudostem, corncob, water hyacinth, and sugarcane bagasse were thoroughly cleaned under running tap water to remove dirt and subsequently dried at 60°C for 4 days to reduce moisture content. The substrates were ground into powder using a blender and sieved through a 600 μm mesh screen to obtain a particle size of 0.25–2.00 mm [37].

All substrates were soaked in water by placing them in baskets covered with a double layer of cheesecloth. Corncob was soaked overnight, while broken rice, banana pseudostem, water hyacinth, and sugarcane bagasse powders were soaked for 1 h. The substrates were gently stirred to remove debris, and the baskets were lifted to drain excess moisture for 30 min. Rice bran was screened using a 600 μm mesh strainer to remove any remaining husk and moistened to the appropriate level before mixing with the other substrates.

Shrimp shell waste, obtained locally, and molasses, procured from local shops, were used as supplements. The shrimp shell waste was cleaned under running tap water, dried at room temperature, crushed, and blended into a fine powder using a blender. The powder was sieved through a 600 μm mesh and stored in a dry place at room temperature. This powder was used as a supplement without demineralization and deproteination. Molasses was used in its received form.

### Solid-State Fermentation

All solid-state fermentations were carried out in 8 × 12-inch polyethylene autoclavable bags containing 100 g of solids substrates mixed with rice bran (8:2 w/w). The initial moisture content of each agricultural waste was adjusted to an optimal value. Afterwards, the mixed substrates were autoclaved at 121°C for 60 min. After sterilization, the autoclaved bags were left over to cool at room temperature. Three milliliters of the conidia broth suspension with initial spore concentration (10^6^ conidia/ml) were aseptically inoculated into each autoclaved moistened sterile substrate [38]. After inoculation, the bags were mixed to ensure uniformity of the fungal inoculum and incubated at the corridor (representing farmers incubated condition) in a perforated box with shelf. Fermentations were carried for 14 days and under the influence of seasonal temperature variation (Nov-Feb). The polyethylene bags were periodically mixed to ensure uniform growth of the conidia within the substrates. Conidia were harvested after every 7 days. All experiments were carried out in triplicates and repeated three times.

### Conidia Production Assessment

The Neubauer chamber (Brand 717810) was used to determine fungal conidia. One hundred grams of the fermented conidiated substrates were mixed with 150 ml of Tween 80 (0.05%). To dissociate the conidiated substrates, the bags were agitated vigorously for 2 min. The conidiated substrates were filtered through a double layer of cheesecloths and vortexed for 1 min before appropriate dilution [37]. All cell counts were performed in triplicate and were related to the dry substrates present in the polyethylene bags at the counting time, following the Equation [39]:

$$\text{Conidia concentration=}\frac{\text{N° of conidia}}{\text{CV. DF}}\cdot \frac{\text{EV}}{\text{SDS}}$$ (1)

where: Conidia concentration (conidia g^-1^ ds); N° of conidia (the counted conidia in the Neubauer chamber at a known dilution); CV, (Neubauer chamber counting volume (ml); DF, (dilution factor of the counting tube); EV,(dilution volume (ml)); SDS, (sample dry substrates (g ds)).

### Single-Factor Experiment

After the selection of the best mixed substrates (white broken rice: rice bran), a screening of single-factor variables, including temperature, initial moisture content, substrate weight, and nutrient supplement (shrimp shell waste and molasses) was carried out to simultaneously optimize the conidia production. The experimental design is detailed in Table 1. Solid mixed substrates without supplementation were used as control. Initial inoculum concentration in these fermentations was 10^6^ conidia/ml, with a temperature of 28 ± 1°C for 7 days (darkness). All experiments were carried out in triplicates and mean values were presented.

### Optimization of SSF Culture Parameters by Response Surface Methodology

BBD of RSM was employed to design four-factor statistical models for establishing variables and interactive effects of the important variables on the fermentation parameters. Each variable was used in three-level according to the results from the single-factor experiment. The BBD was employed using Design expert software (12.0.3) and the interaction levels were presented in Table 2. To predict the optimum culture conditions for the conidia production, a quadratic model was adopted to examine the effects of independent variables to the response.

$$Y=\beta_{0}+\beta_{A}A+\beta_{B}B+\beta_{C}C+\beta_{D}D+\beta_{AB}AB+\beta_{AC}AC+\beta_{AD}AD+\beta_{BC}BC+\beta_{BD}BD+\beta_{CD}CD+\beta_{AA}A^{2}+\beta_{BB}B^{2}+\beta_{CC}C^{2}+\beta_{DD}D^{2}$$ (2)

where Y is the response surfaces for conidia production; β represents regression coefficient; β_0_ is the constant term (intercept); β_A_, β_B_, β_C_, and β_D_ are the linear terms coefficients; A, B, C, and D represent the coded levels of the independent variables; β_AA_, β_BB_, β_CC_, and β_DD_ represent the coefficients of the quadratic terms; and β_AB_, β_AC_, β_AD_, β_BC_, β_BD_, and β_CD_ represent the coefficients of the cross-product parameters.

The adequacy of the models is checked by model analysis and *R*^2^ analysis; *F*-value is inspected to find out the significance of all the fitted equations. The corresponding coefficients of variables, interaction variables, and response surface graphs were drawn by Design Expert software. The optimum values of the selected variables were obtained by solving the regression equation and by analyzing the response surface plots. Based on the optimization model, a new fermentation procedure was implemented using Design Expert software to verify and solve the regression equation with the optimized culture parameters, the fermentation was performed in triplicate at the ideal conditions.

### Performance Indices and Enzyme Activity

Based on the optimized model verification results, a new SSF experiment was performed to determine the effects of the optimized model on performance indices (number of conidia, weight of conidia, water content, consumed substrates and conidia germination) and cuticle-degrading enzymes (chitinase, lipase, and protease) activity of the fermented conidia. Dry aerial conidia of the MN031-Mt 46 and its corresponding Wt isolates were used to assess the performance indices. At harvest, fermented conidiated substrates from each bag were dispensed into a sterile aluminum foil and allowed to dry for 7 days at 28 ± 1°C. The dried fermented conidia were harvested by gently sifting through a mesh sieve (295 μm mesh size). Three replicated production sets were run for each optimized validated quadratic model and substrates without optimized validated variables were used as a control. Fermented conidia powder from each polyethylene bag was taken to estimate the following performance indices (i) mean number of fermented conidia per gram of powder (ii) weight of fermented conidia powder per kg of the substrate, (iii) percentage water content (based on weight loss of 1 g powder dried at 120°C for 2 h), (iv) percentage consumed substrate in each bag (based on the weight of the dry fermented substrate before inoculation and that of the dry fermented substrate residues immediately after harvesting the conidia) [10]. Conidial viability of the entomopathogens was also evaluated by germination test on SDA medium through incubating at 28°C for 24 h adopting the method of [40]. Percent germination of conidia was determined after having treated the medium with 70% ethanol to halt germination [41]. The number of germinated conidia was detected based on the formation of conidia germ tubes under 40× magnification of a compound microscope. The percentage germination was calculated using the following formula.

$$G=\frac{TC-NG}{TC}\cdot 100$$ (3)

where, present of G: germinated conidia; NG: non-germinated conidia and TC: total count of conidia.

### Enzymatic Assays

**Preparation of culture filtrate and reagents.** The colonized fermented conidia based on the optimization results were used in enzyme assays. Thirty grams of the fermented conidia were aseptically transferred into a 500 ml Erlenmeyer flask containing 300 ml of sterile distilled water. The flasks were stirred at 1,000 rpm using a magnetic stirrer (Heidolph, Model MR 3001) for 10 min. Afterwards, the mixture was filtered through a double layer of cheesecloth and the suspensions were centrifuged for 10 min at 9,000 rpm using a 50 ml tube to obtain the supernatant. The crude filtrate was used in the enzymatic determination assays. A modified protocol of Murthy and Bleakley [42] was used for the preparation of colloidal chitin. The prepared colloidal chitin was covered with aluminum foil and sterilized by autoclaving at 15 psi, 15 min, 121°C. The autoclaved colloidal chitin was stored at 4°C to be used in moist form, based on the protocol of Gómez *et al*. [43].

### Enzyme Index (EI) Determination

The *protease index* was assessed by measuring the clear hydrolysis zone in casein hydrolysis agar, which was composed of 1 g KH_2_PO_4_, 0.5 g KCl, 0.4 g MgSO_4_·7H_2_O, 0.1 g CaCl_2_·2H_2_O, 25 ml of 15% powdered skim milk, 10 g glucose, 12 g agar, and 1,000 ml distilled H_2_O[44].

*Chitinase index* was determined using colloidal chitin agar prepared based on the modified protocol of Gómez *et al*. [43] and Hsu and Lockwood [46]. The colloidal chitin agar medium composed of 1 g (NH_4_)_2_SO_4_, 1 g K_2_HPO, 0.5 g KCl, 5 g NaCl, 0.5 g MgSO_4_, 0.01 g FeSO4, 20 g agar, 5% moist colloidal chitin and 1,000 ml distilled H_2_O [47].

*Lipase index* was determined according to [48] with some modifications. The basal medium had the following composition (g/l):1.2 g NaH_2_PO_4_, 0.3 g MgSO_4_·7H_2_O, 2 g KH_2_PO_4_, 0.25 g CaCl_2_, 0.003% NaCl, 2% agar, 1%(NH_4_)_2_SO_4_, 0.15% Tween 80 and 2% olive oil. Enzyme index screening method was performed by adding 100 μl of the crude enzyme filtrate into a 10-millimeter well at the center of each medium plate and plates were incubated at 28°C for 10 days in the dark [45]. The enzyme index (EI)was determined for each enzyme and calculated using the following formula [49]. All experiments were performed in triplicates.

$$\text{Enzyme Index}=\frac{\text{Hyrolysis zone diameter}}{\text{Colony diameter}}$$ (4)

### Enzyme Activity Determination

The *proteolytic activity* of the diluted crude enzyme extract was assayed using casein sodium salt from bovine milk (Fluka Biochemika.) adopting the conditions described by Cupp-Enyard [50], with some modification. Twenty-five μl of diluted samples was mixed with 130 μl of 2% casein from bovine milk. The reaction was incubated for 10 min and stopped by adding 130 μl of 110 mM trichloroacetic acid followed by incubation for 20 min. The supernatant was mixed with 625 μl of 500 mM sodium carbonate and 125 μl of 0.5 M Folin-Ciocalteu. Absorbance was measured at 660 nm. One unit of protease activity (U g^-1^ ds) was defined as the amount of enzyme that released 1 μmol of tyrosine per minute.

*Chitinolytic* activity was determined using colloidal chitin adopting the condition as described by Vahed *et al*. [51] with some modification. Diluted samples (1,000 μl) were mixed with 2,000 μl of 5 % moist colloidal chitin [46, 52]. The reducing sugar released by the reaction mixture (N-acetyl glucosamine) was quantified by DNS method [53] with a N-acetyl glucosamine prepared calibration curve (0–5 mg/ml). Supernatant absorbance was measured at 540 nm. One unit of chitinase activity (U g^-1^ ds) was defined as the amount of enzyme that released 1 μmol of N-acetyl glucosamine per minute.

*Lipolytic* activity was performed using *p*-nitrophenyl palmitate (*p*-NPP) as substrate according to the method described by Pencreac'h and Baratti [54] with some modification. The assay mixture was prepared by adding 90 μl of 8.25 mM *p*-NPP in isopropanol and 810 μl of solution containing 50 mM Tris-HCl buffer (pH 8.0) with 0.494%Triton X-100 and 0.12% arabic gum, followed by pre-incubation at 40°C for 10 min. The enzymatic reaction was then initiated by adding 100 μl of appropriate diluted lipase sample (culture supernatant) and proceeded at 40°C for 10 min. The amount of *p*-nitrophenol released was measured at 420 nm. One unit of the lipase activity (U g^-1^ ds) was defined as the amount of enzyme that released 1 μmol *p*-nitrophenol per minute. Enzyme free reaction was used as the control. All enzyme activities were measured using 96 well Thermoscientific spectrophotometer (Multiskan TM SkyHigh, Model 1550).

### Statistical Analysis

The concentration of conidia per gram of dry substrates in all fermentation was compared using analysis of variance (*p* < 0.05) with the Turkey HD test. RSM optimization results were log transformed before analysis on Design Expert (12.0.3). A Student's t-test was used to compare the means of the actual and predicted RSM optimized validated variables. IBM Corp [55] was used for data analysis, while GraphPad Prism [56] was used to construct graphical representations.

## Results

### Screening of Solid Substrates

**Fig. 1:**
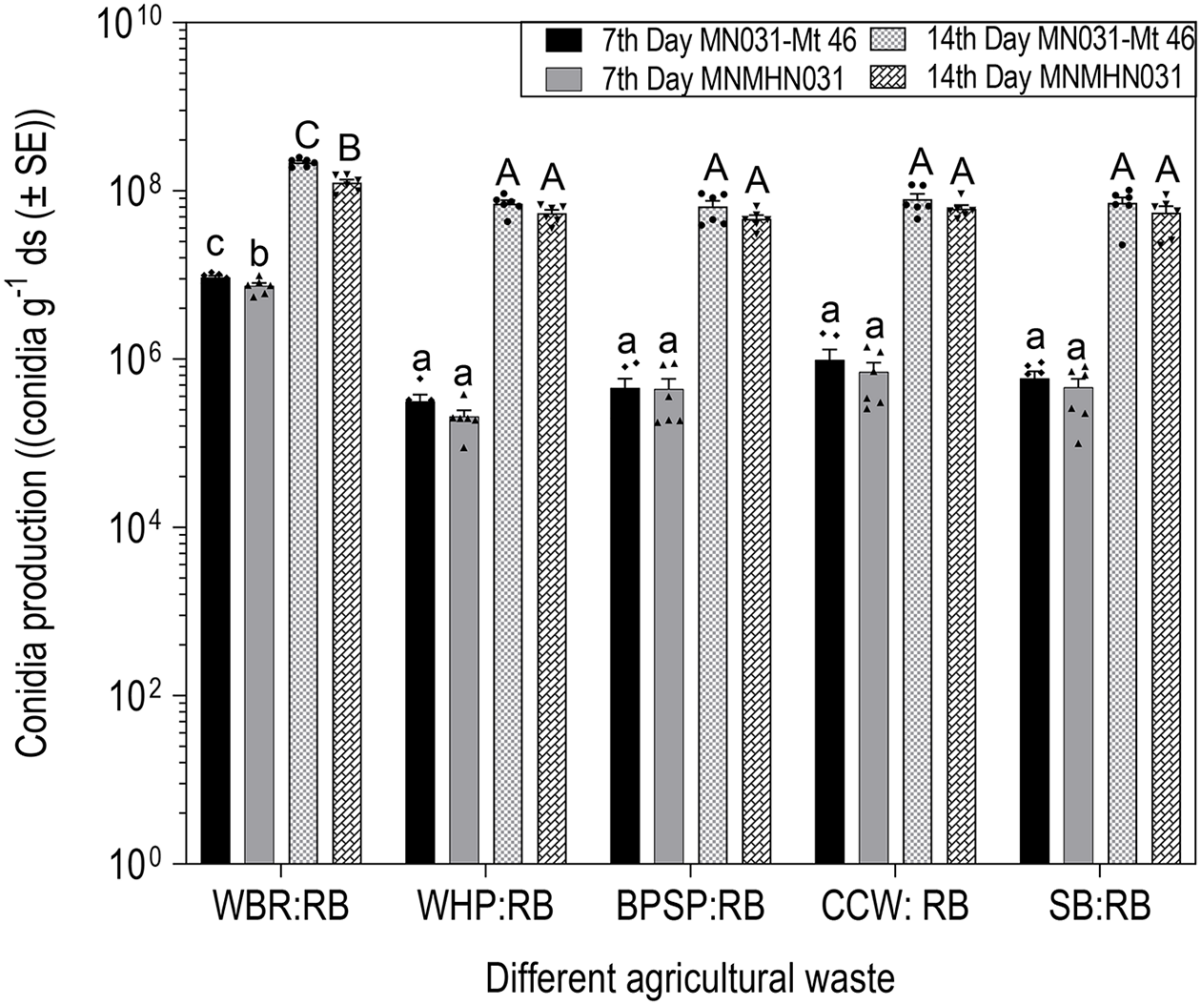
Conidia production of mutant *M. koreanum* (MN031-Mt 46) and wild-type (MNMHN031) on different mixed solid agricultural waste. Different agricultural waste tested included WHP: water hyacinth powder, BPSP: banana pseudostem powder, SB: Sugarcane bagasse, CCW: corncob waste, WBR: broken rice, RB: rice bran. Error bars are standard errors and mean at 95% Cl. Different letters indicate statistically significant differences (*p* > 0.05, Turkey HD test). Lowercase (7^th^ day) and uppercase (14^th^ day) letters represent statistical differences between treatments. Data points indicate the means of three replicates.

Different agricultural wastes (white broken rice, water hyacinth, banana pseudostem, corncob, and sugarcane bagasse) mixed with rice bran (8:2) were used to evaluate conidia production of entomopathogenic fungus *M. koreanum* MN031-Mt 46 and MNMHN031 in SSF. Among the mixed substrates screened, the result indicated that both MN031-Mt 46 and MNMHN031 isolates had the highest conidia production on mixed white broken rice (WBR): rice bran (RB) substrate followed by corncob waste (CCW) : RB. Conidia production reached 9.30 × 10^6^ and 7.4 × 10^6^ conidia g^-1^ ds on the 7^th^ day of fermentation, then the production significantly increased reaching 2.18 × 10^8^ and 1.25 × 10^8^ conidia g^-1^ ds on the 14^th^ day of fermentation (Fig. 1). CCW: RB produces the second highest conidia production for both isolates reaching 9.82 × 10^5^, and 7.05 × 10^5^ conidia g^-1^ ds on the 7^th^ day of fermentation, increasing to 7.98 × 10^7^ conidia g^-1^ ds on the 14^th^ day of fermentation. Lower conidia production was overall achieved on water hyacinth (WHP) : RB for both MN031-Mt 46 and MNMHN031 producing 3.16 × 10^5^ and 2.0 × 10^5^ conidia g^-1^ ds on the 7^th^ day of fermentation (Fig. 1), while banana pseudostem powder (BPSP) : RB produced the lowest conidia at 4.67 × 10^7^ conidia g^-1^ ds for MNMHN031 and 6.50 × 10^7^ conidia g^-1^ ds for MN031-Mt 46 on the 14^th^ day of fermentation (Fig. 1). Statistically, WBR: RB conidia productions were significantly different (F = 152.02, df = 8, *p* < 0.001; F = 31.80, df = 8, *p* < 0.001) compared to other substrates with high conidia yield. No significant differences in conidia production from the wild type (MNMHN031) and the mutant (MN031-Mt 46) isolates fermented on WHP, BPSP, CCW and SB mixed with rice bran was observed at both 7 and 14 days of incubation. However, a significant increase in conidia production for both isolates was observed on WBR: RB.

### Single-Factor Experimental Results

**Fig. 2:**
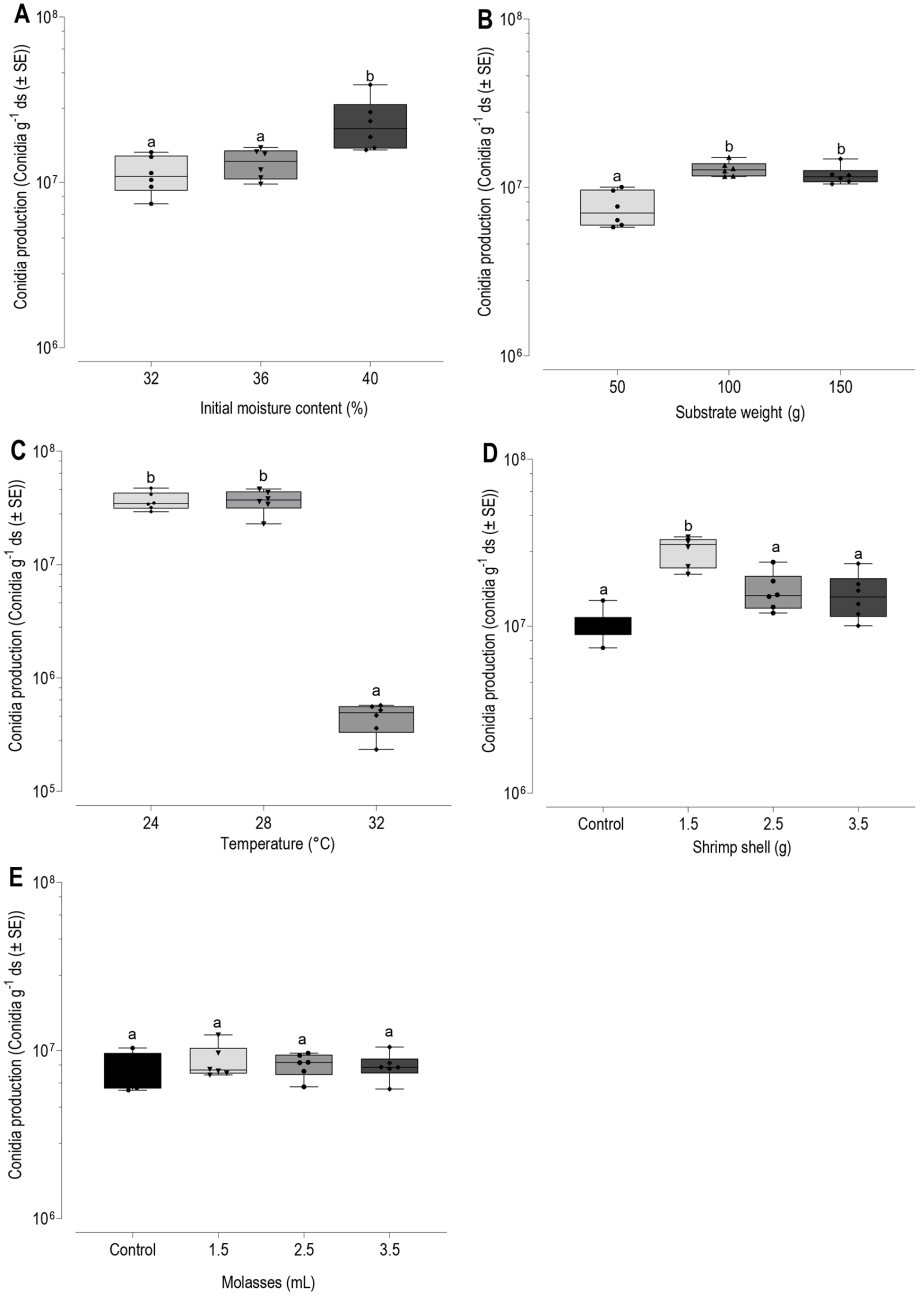
Single-factor at a time optimization of the fermentation process of five factors on conidia production of *M. koreanum* (MN031-Mt 46). (**A**) Moisture content, (**B**) Substrate weight, (**C**) Temperature. (**D**) Shrimp shell waste. (**E**) Molasses supplementation. The same letter on the box plots indicates statistically insignificant variation (*p* > 0.05, Turkey HD test) among the treatments.

In this study, a range of single factor (initial moisture, substrate weight, temperature, nutrient supplements (shrimp shell waste and molasses)) were evaluated for the optimum conidia production by MN031-Mt 46 on WBR: RB that produced the highest conidia production in an earlier agricultural waste test. The result from these single-factor experiments provides the basis for selecting center points for RSM optimization design. Results of different initial moisture content (32, 36, 40%) evaluated for conidia production, indicated that the conidia production was 1.13 × 10^7^ conidia g^-1^ ds at 32%, which slightly increased to 1.31 × 10^7^ conidia g^-1^ ds at 36%; then rapidly increased to its highest value of 2.3 × 10^7^ conidia g^-1^ ds at 40% initial moisture content. Significant differences (F = 8.02 _2, 15_, *p* = 0.0043) in conidia production were observed as the moisture increased from 32 to 40% (Fig. 2A). As depicted in Fig. 2B, MN031-Mt 46 exhibited conidia production of 7.5 × 10^6^ conidia g^-1^ ds at 50 g, which was significantly lower (F = 19, _2, 15_, *p* < 0.0001) than 1.28 × 10^7^ conidia g^-1^ ds and 1.16 × 10^7^ conidia g^-1^ ds at 100 g and 150 g, respectively. Temperature significantly (F = 69.89 _2,15_, *p* < 0.0001) affected conidia production of MN031-Mt 46. Fig. 2C shows that conidia production was 3.65 × 10^7^ conidia g^-1^ ds at temperature 24°C which was slightly increased to 3.68 × 10^7^ conidia g^-1^ ds at 28°C. However, a decline in conidia production (4.5 × 10^5^ conidia g^-1^ ds) was observed as the temperature increased to 32°C. As depicted in Fig. 2D, the addition of shrimp shell waste had significant effect on conidia production (F = 18.22 _3,20_, *p* < 0.0001) with the most effective supplementation level at 1.5 g (2.87 × 10^7^ conidia g^-1^ ds) compared to the control (without supplementation) at 1.00 × 10^7^ conidia g^-1^ ds. However, addition of molasses as supplement had no significant effect on conidia production (F = 0.27 _3,20_, *p* = 0.84). Fig. 2E shows that the conidia production reached 8.65 × 10^6^ conidia g^-1^ ds at 1.5 ml of molasses, with conidia production declining to 8.07 ×106 conidia g^-1^ ds as the volume of the molasses increased to 3.5 ml. Overall, conidia production was higher in the molasses-supplemented medium compared to the control.

### Response Surface Optimization Result for Conidia Production

Response surface methodology is a widely used technique for optimizing culture parameters and providing optimal conditions through mathematical and statistical analysis. Based on the findings from one-factor-at-a-time experiments and the effects of nutrient supplements on conidia production, the Box-Behnken design was employed to determine the optimal culture parameters for conidial production. Table 3 presents the results of 29 experimental runs, detailing the different culture parameter levels and the predicted and observed outcomes. The observed values for the conidial production of the mutant isolate *M. koreanum* (MN031-Mt 46) ranged from 2.68 to 8.37 (log conidia g^-1^ ds) under various experimental conditions. The following quadratic equation represents the regression model predicting conidia production based on the data analysis

$$Y=+7.37-1.16A+0.09B-0.08C+1.86C+0.50AB-0.63AC+0.20AD-0.54BC+0.13BD-0.45C-1.49A^{2}-0.37B^{2}-0.37C^{2}-0.87D^{2}$$

In this study, the yield (Y) of conidia (log conidia g^-1^ ds) was determined by the variables (A), (B), (C), and (D). The ANOVA results (Table S2) showed a model *F*-value of 60.62 with a *p*-value (*P* < *F*) of 0.0001, indicating that the regression model used in the experiment is statistically significant. The quadratic terms of temperature (A^2^), initial moisture content (B^2^), shrimp shell (C^2^), and incubation time (D^2^) significantly influenced conidia production (Table S1). Additionally, the ANOVA revealed that the interactions between temperature and moisture content (AB), temperature and shrimp shell (AC), temperature and incubation (AD), moisture content and shrimp shell (BC), as well as shrimp shell and incubation time (CD) significantly affected conidia production (*p* > 0.05). The R^2^ value of 0.9838 demonstrated that the independent variables accounted for 98.38 % of the variation in conidia production of MN031-Mt 46. The adjusted R^2^ value of 0.9675 was also high, indicating the model’s significance in navigating the experimental design space. The coefficient of variation (CV) value of 5.02%was reasonably low, suggesting that the studies were efficient and accurate.

**Fig. 3:**
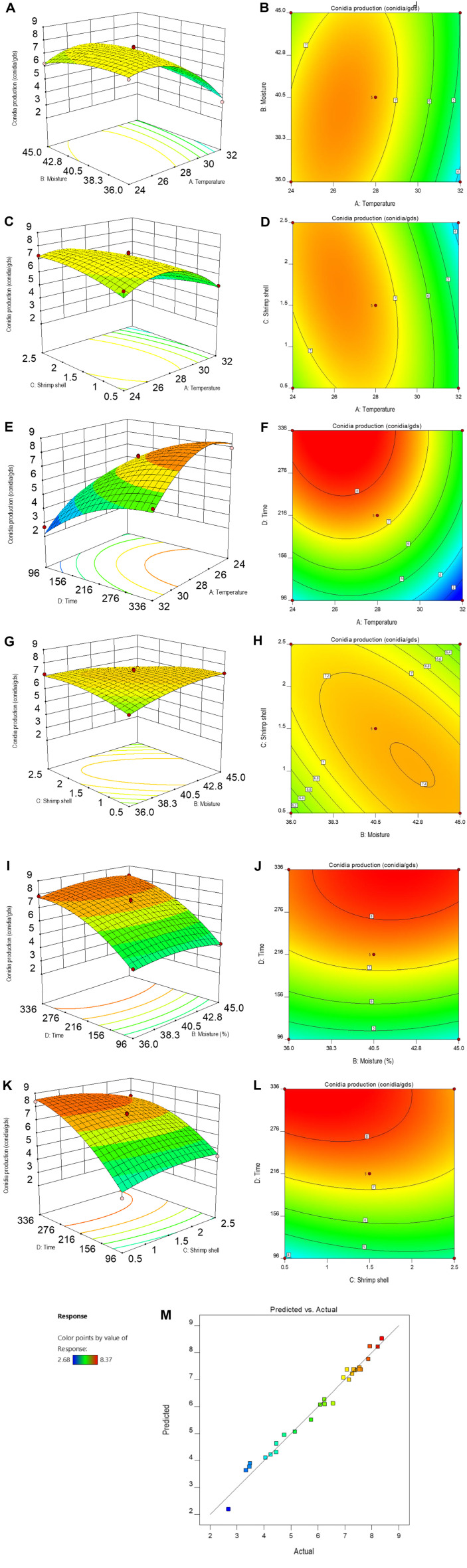
Response surface experiment results of conidia production of mutant isolates of *M. koreanum* (MN031-Mt 46). (**A–L**) 3D response surface map and 2D contour plots corresponding to the interaction between different variables on the conidia production of MN031-Mt 46 at *p* < 0.05. (**A–B**) moisture level vs temperature, (**C–D**) shrimp shell vs temperature, (**E–F**) incubation vs temperature, (**G–H**) shrimp shell vs moisture level, (**I–J**) incubation time vs moisture levels, (**K–L**) incubation time vs shrimp shell and (**M**) Correlation of actual and predicted values of MN031-Mt 46 conidia production (conidia/g ds).

The 3D graph and 2D contour plots (Fig. 3A and 3B) from the response surface illustrate the interaction between moisture levels and temperature on conidia production. The response surface results indicated that temperature had a more significant effect on conidia production compared to other variables. The curves show that moisture content at both lower and higher levels decreased conidia production, while a significant increase in conidia production was observed at the middle of the curve. This demonstrates that higher moisture levels increase substrate thickness, significantly reducing the surface area and affecting conidia growth. An increase in temperature from 24°C to 28°C resulted in higher conidia production, followed by a gradual decline as the temperature further increased from 30°C to 32°C.

Fig. 3C and 3D illustrates the interaction between shrimp shell waste and culture temperature on conidia production. Production decreased rapidly with increasing temperature and low amounts of shrimp shells. Maximum conidia production was observed at mid-temperature and higher amounts of shrimp shell waste, indicating that temperature has a more distinct effect on conidia production than shrimp shell waste at low levels.

No significant difference was observed for the interaction between incubation time and temperature (Fig. 3E-3F, Table S1). However, higher conidia production was achieved by increasing the incubation time and decreasing the temperature. The interaction between shrimp shell waste and moisture is shown in Fig. 3G–3H, where optimal conidia production was attained with high amounts of shrimp shell waste and higher moisture levels. Conidia production decreases rapidly with reduced shrimp shell and moisture content or excessive levels of shrimp shell and moisture content.

The interaction between incubation time and moisture exhibited no significant difference (Fig. 3I–3J, Table S1). The result indicated that extending the incubation time was associated with increased conidia production for all moisture levels. Fig. 3K–3L shows the interaction between incubation time and shrimp shell waste on conidia production. Data from the response surface and contour plots indicated that extending the incubation time and the amount of shrimp shell around 1.5% (w/w) increased conidia production. Higher amounts of shrimp shell gradually decreased conidia production following prolonged incubation. Fig. 3M shows a high correlation between actual and predicted conidia production, depicting a linear distribution and indicating a well-fitted model.

**Validation of RSM optimized fermentation test.** Validation of the RSM optimized fermentation results was carried out according to the optimization model. Using Design Expert Software, the optimal levels for each variable in the substrates were determined: 26.19°C incubation temperature, 39.76 % initial moisture content, 1.45% (w/w) shrimp shell waste, and 301.87 h of incubation time. When MN031-Mt 46 conidia was fermented with this optimized medium, conidia production reached 8.45 log conidia g^-1^ ds, closely matching the predicted results of 8.44 log conidia g ¹ ds. The difference between validated results (actual) and model predicted result is not statistically significant (*p* < 0.05) (Table S3). This confirmed the validity and feasibility of the optimal model with a considerable reduction in incubation time and increase in conidia production.

**Fig. 4:**
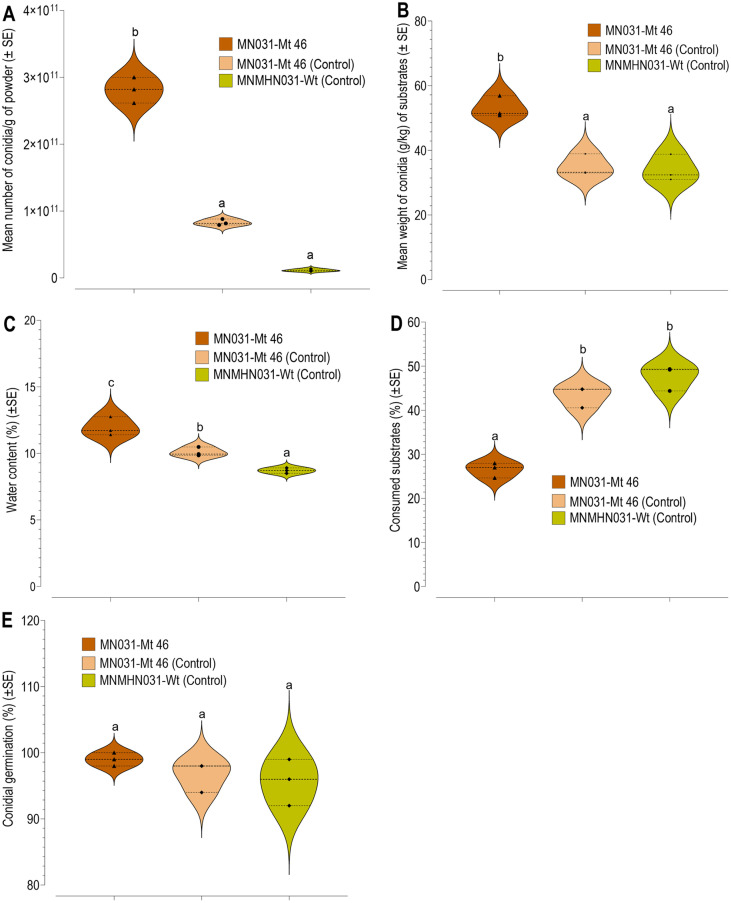
Performance indices results based on RSM-optimized substrates for *M. koreanum* isolate (MN031-Mt 46) and wild-type (MNHMN031). (**A**) Mean number of conidia/g of powder. (**B**). Mean weight of conidia/kg of powder. (**C**) Percentage water content of the conidia. (**D**) Percentage consumed substrates. (**E**) Conidia germination. Different letters above error bars indicate a significant difference across the treatment. The same letter on the violin plots indicates statistically insignificant variation (*p* > 0.05, Turkey HD test) among the treatments (optimized and control fermentation).

**Fig. 5:**
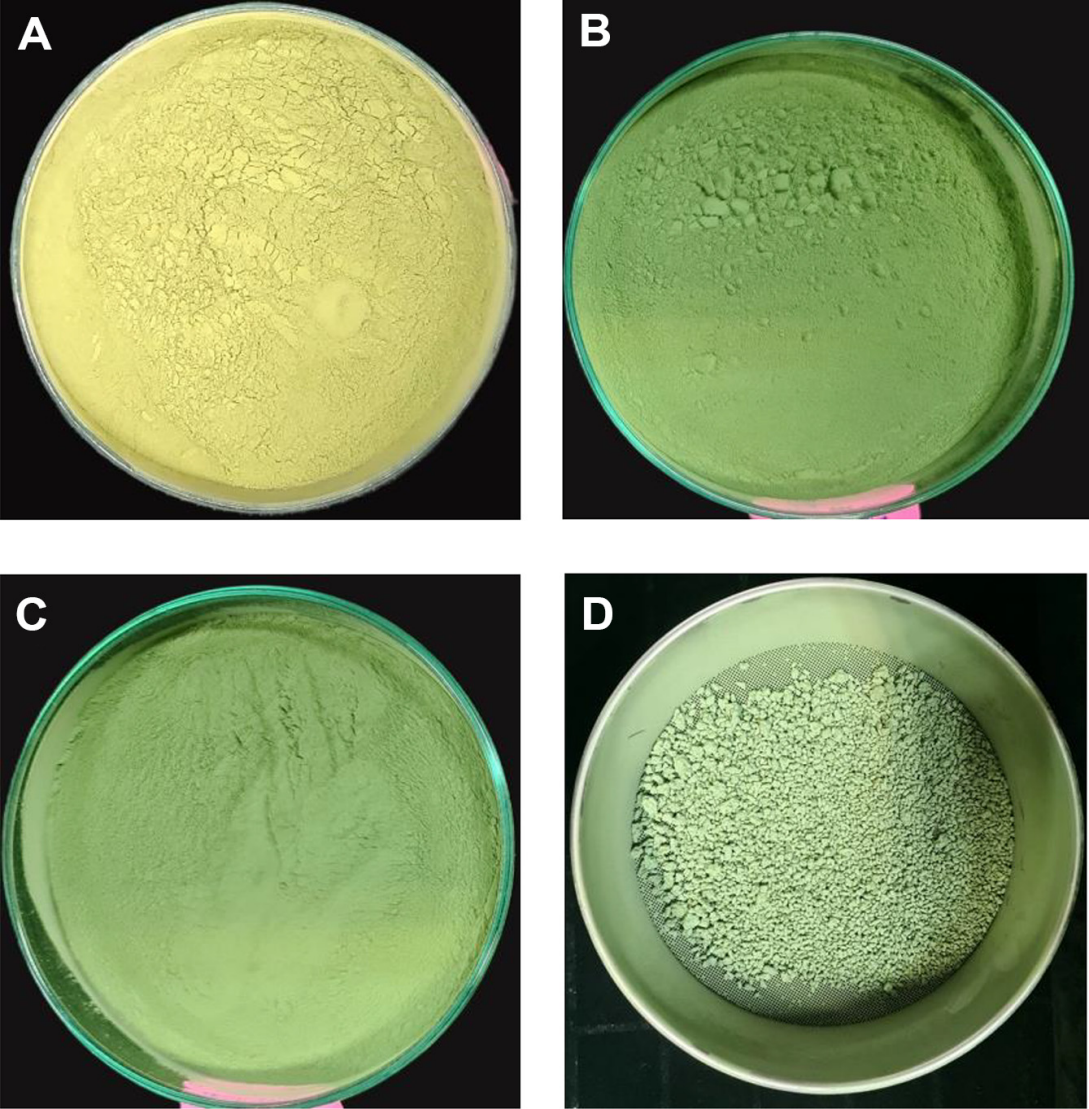
Results of performance indices. (**A**) Dry aerial conidia of M. korenum (MN031-Mt 46) based on optimized medium variables, (**B**) MN031-Mt 46 (control), (**C**) Wild-type MNMHN031(control) and (**D**) The consumed residues after conidia harvest.

**Performance indices and cuticle-degrading enzyme activity.** The number of conidia per gram of powder and weight of conidia harvested per kilogram of mixed substrates (broken rice, rice bran, and shrimp shell) were significantly higher for MN01-Mt 46 (optimized) compared to MN031-Mt 46 and MNMHN031-Wt (control)(Fig. 4A and 4B). All treated parameters had a significant impact on the yield of conidial substrate. The validated substrates for MN031-Mt 46 produced the highest conidia powder yield at 53.04 ± 3.35 g/kg of substrate, compared to the control MN031-Mt 46 and Wt (MNMHN031), which yielded 35.03 ± 3.35 g/kg and 34.06 ± 4.15 g/kg, respectively (F = 25.85, df = 2, *p* < 0.001) (Fig. 4B). The percentage water content was significantly higher for the validated cultured substrates compared to the control (F = 36.46, df = 2, *p* < 0.001) (Fig. 4C). Additionally, the weight of substrate residue after conidia harvest was significantly lower in the validated substrates than in the control (F = 66.76, df = 2, *p* < 0.001) (Fig. 4D). The viability of conidia powder from RSM model validation parameters and the control, assessed at 24 hours, showed that the percentage viability reached 90% for all validated and unvalidated process parameters, regardless of the process. No statistically significant differences were observed among the treatments (Fig. 4E). Different color pigmentation was observed on substrates supplemented with shrimp shell waste. Substrates without shrimp shells showed lighter green pigmentation, whereas substrates with shrimp shells exhibited olive green. (Fig. 5).

### Cuticle-Degrading Enzyme Activity

**Fig. 6:**
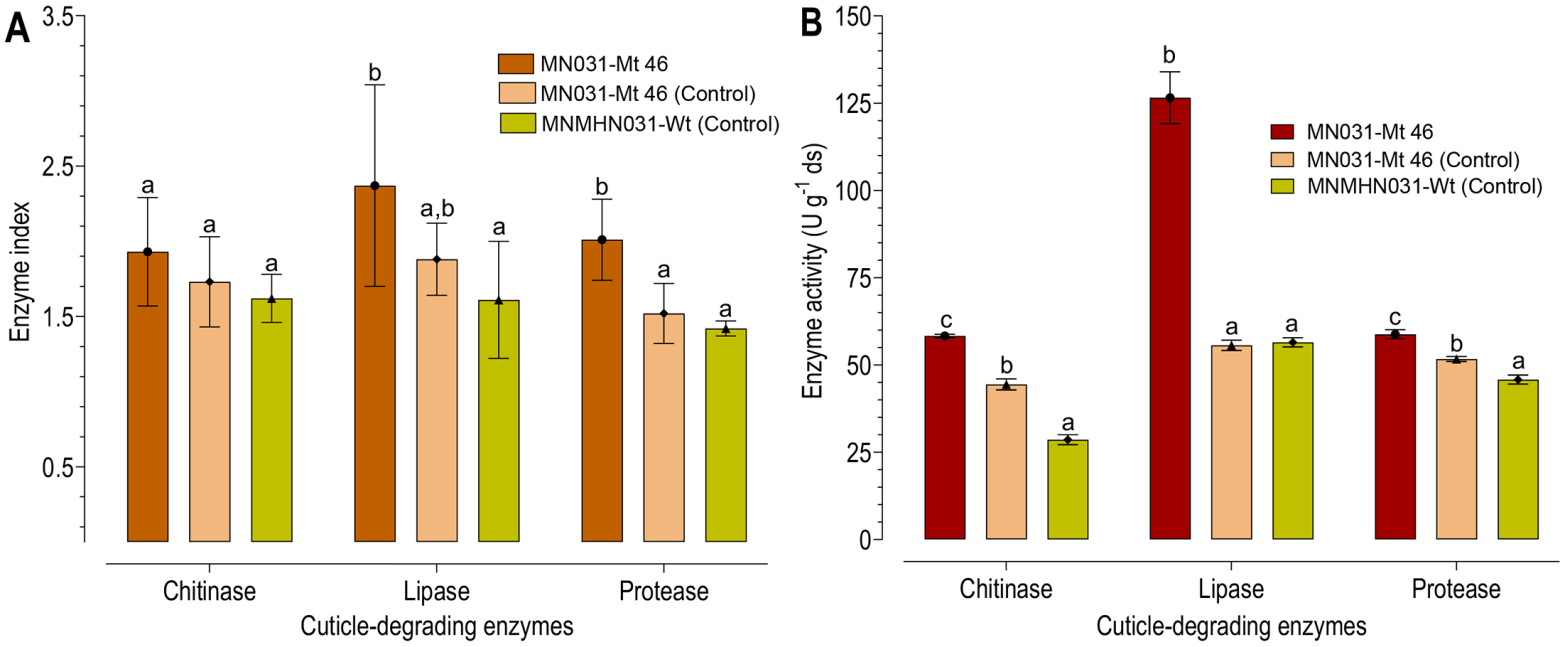
Cuticle-degrading enzyme activity of *M. koreanum* fermented conidia obtained based on RSMoptimized and control parameters. (**A**) Enzyme index, (**B**) Enzymatic activity (U g^−1^ ds). The same letter indicates statistically insignificant variation (*p* > 0.05, Turkey HSD test) among each enzyme test.

**Enzyme index.** All fungal isolates cultured using RSM-optimized fermented substrates showed significant qualitative enzyme activity compared with the control (unoptimized). Protease activity ranged from 1.42 ± 0.05 to 2.01 ± 0.27, chitinase from 1.62 ± 0.16 to 1.93 ± 0.36, and lipase from 1.61 ± 0.39 to 2.37 ± 0.67 (Fig. 6A) were observed. The RSM-optimized fermented substrates displayed the highest average indices for lipase (2.37), protease (2.01) and chitinase (1.93), with no significant differences observed in the qualitative chitinase index between the RSM-optimized and the control (Fig. 6A).

**Enzyme activity.** The mutant MN031-Mt 46 produced higher levels of cell-wall degrading enzymes, including protease, chitinase, and lipase, when using RSM-fermented model substrates. The addition of shrimp shell waste to the substrates resulted in significantly higher enzymatic activity compared to the control (Fig. 6B). Protease activity was higher in RSM fermented model substrates compared with the control, with values ranging from 45.83 ± 2.23 to 58.78 ± 2.20 U g^-1^ ds. Chitinase activity was notably elevated in substrates supplemented with shrimp shell waste compared with the control. The chitinase activity on RSM-fermented model substrates range from 28.69 ± 2.49 and 58.32 ± 0.78 U g^-1^ ds. Among the enzymatic activities evaluated, *M. koreanum* MN031-Mt 46 on RSM-fermentated model substrates produced a higher level of lipase activity compared with the control (F = 17, df = 2, *p* < 0.001). The lipase activity ranges between 56.57 ± 2.26 and 126.57 ± 6.47 U g^-1^ ds (Fig. 6B).

## Discussion

This study assessed various mixed solid agricultural wastes as potential less expensive substrate for conidia production of *M. koreanum* isolates (MN031-Mt 46 and MNMHN031). All substrates screened showed potential for significant conidia production, even though differences in the quantity of conidia produced was observed among the substrates. The findings indicate that a mixture of white broken rice and rice bran was considerably better substrates for conidia production of *M. koreanum* isolates. The nutritional composition of solid substrates significantly influences conidia production [57, 58]. Lignocellulosic wastes (banana pseudostem, corncob, water hyacinth and sugarcane bagasse) consist of three major components, including cellulose, hemicellulose, and lignin [59-62]. To improve waste quality, the addition of rice bran into the lignocellulosic solid substrates greatly influenced their conidia production capacity. Rice bran contains 50% carbohydrates, 20% fat, 15% protein, and 15% dietary fiber [63, 64]. Rice bran protein is high in lysine and low in glutamic acid compared to rice and wheat [65, 66]. The substantial increase in conidia production from the mixture of lignocellulosic waste and rice bran in this study is attributed to these nutritional factors. The interaction between the mixture of these two solid substrates may enhance conidia production of *M. koreanum* isolates under a stable temperature condition. In comparison to the lignocellulosic waste, white broken rice is rich in starch, fiber, and other higher quantities of minerals and vitamins [67]. Quintela [68] reported that the production of conidia of *M. anisopliae* on coarse grain is significantly greater than on whole grain. The physical structure of solid substrates also influences conidia production, with good aeration between particles being essential [69, 70]. This is relevant to our study in which white broken rice was selected instead of whole white rice grain. Dallastra *et al*. [71] reported a similar conidia concentration (10^8^ conidia/ml) using broken rice and wheat bran in *M. anisopliae*. However, our study’s conidia production using a mixture of broken rice and rice bran exceeded the yield (2.22 × 10^6^) reported by Bich *et al*. [72] using broken rice alone. The present study suggests that the nutritional content of a mixed solid substrate (white broken rice and rice bran) is more favorable for conidia production than lignocellulosic waste, even though the lignocellulosic waste exhibited remarkable conidia production.

In this study, the single factor optimization results revealed that moisture content of a substrate plays a key role in conidia production, with production increasing at higher moisture levels and decreasing at lower levels, reaching an optimum production at 40 % moisture content. This aligns with the findings by da Cunha *et al*. [73] and Bück *et al*. [74], who observed that low moisture levels reduce microbial growth and substrate utilization. The moisture content of the substrate significantly influences SSF processes, varying based on the substrate’s nature, the desired product, and the requirements of the microorganism [75]. Cavalcante *et al*. [76] and Pang *et al*. [77] reported that moisture content causes the substrate to swell, which facilitates fungal nutrient absorption for growth and metabolic activities. Manpreet *et al*. [78] identified the optimal substrate particle size as between 1 mm and 1 cm, effectively balancing nutrient accessibility and oxygen availability. Similarly, our findings show that a mixture of broken rice and rice bran (600 μm–3 mm) at 100–150 g significantly produced the highest conidia. It is suggested that the addition of rice bran likely increases substrates porosity, enhancing conidia formation. Smaller particle sizes increase the surface area available for microbial access, enhancing heat and oxygen transfer [79, 80].

Temperature is crucial for microbial growth and sporulation. Bayissa *et al*. [81] and Mweke *et al*. [82] reported that *M. anisopliae* *grew* well between 15 and 30°C, with an optimal range of 20–30°C. Muazu *et al*. [35] found that gamma-induced mutant isolates of *M. koreanum* could grow in a wide temperature range of 28–42°C. Rangel *et al*.[83] observed that elevated temperatures inhibit the conidial germination of *Metarhizium* strains, which could explain the decline in conidia production of *M. koreanum* in this study as temperature increased from 28 to 32°C.

Afandhi *et al*. [84] reported that enriching rice bran with cricket powder supported the mass production of virulent *B. bassiana*, aligning with our findings that supplementing mixed substrates of broken rice and rice bran with shrimp shell waste enhanced the growth and sporulation of *M. koreanum* MN031-Mt 46 isolate compared with the control. However, molasses as a supplement did not increase conidia production. Supplements such as Torula yeast extract or sugarcane molasses were reported to fulfill the nutrient requirements of some agricultural substrates and increase conidia production [85-88].

The primary focus of developing biopesticides from agricultural waste is to lower production costs and enhance yield. In this study, the experimental results revealed that the optimized medium for conidia production using RSM model for *M. koreanum* MN031-Mt 46 included 26.19°C incubation temperature, 39.76% initial moisture content, 1.45% (w/w) shrimp shell waste, and 301.87 h of incubation time. The conidia production of *M. koreanum* MN031-Mt 46 reached 8.45 log conidia g^-1^ ds after 301.87 h (12.58 days) in the optimized fermentation model with a reduction in incubation time. Sachdev, Singh [89] achieved maximum spore production of *T. lixii* TVR1 (1.91 × 10^8^ spores g^-1^) under specific fermented conditions at 30°C with 68.87% moisture content and 31 days of incubation by RSM. Prakash *et al*. [87] found optimal spore production using SSF for rice at 22.24% moisture content with 1.45% yeast extract. Their results indicated that the addition of yeast extract at 1.45% increased conidia production of *M. anisopliae*. Zhang *et al*. [37] reported an optimal culture environment of 29.1°C, 56.13% initial moisture content, and 7.51 g material weight using cassava peel, achieving an optimal spore production (9.31 × 10^9^ and 9.91 × 10^9^ spores g^-1^). However, RSM optimization of *M. koreanum* MN031-Mt 46 mutant isolate showed promising conidia production (8.45 log conidia g^-1^ ds) under different fermentation conditions probably attributable to the use of different substrates and supplements including shrimp shell waste.

In terms of *M. koreanum* MN031-Mt 46 performance indices, the results depicted that the RSM optimized fermentation model had a significant impact on the indices. The RSM optimized fermentation model outperformed the controls, producing the highest conidia yield, consuming less substrate, and displaying high moisture content, indicating the isolates’ colonization ability in the substrates. Tumuhaise *et al*. [10] reported a similar substrate consumption pattern of 32.8%, and Agbessenou *et al*. [90] reported a value close to 25%, which aligns with our findings of 26–47% substrate consumption rates. Machado *et al*. [91] and Mascarin and Jaronski [11] reported that substrates and additives used in a fermentation process must provide a high availability of nutrients over a large surface area to promote germination and conidia formation. Our finding indicates that the percentage conidia germination reached 90% with the optimized fermentation model, which supports the entomopathogenic efficiency of the fungus.

The optimized fermentation model significantly influenced the production of cuticle-degrading enzymes (protease, chitinase, and lipase) in the *M. koreanum* MN031-Mt 46 isolate. The results showed that the addition of shrimp shell waste to the substrates led to a significant increase in enzymatic activity compared to the control. This aligns with the report of Mejía *et al*. [34] that enzymatic activity is affected by the nitrogen and micronutrient supplementation. The increased enzymatic activity observed in the RSM-optimized fermentation model substrates suggested that these activities were influenced by the four variables (temperature, moisture, shrimp shell, and incubation time) and their interactions. Increased enzyme synthesis was observed in MN031-Mt 46 compared to MNMHN031 from unoptimized fermentation. This increase may be attributed to gamma mutagenesis of the Wt isolate from previous work [35]. Aita *et al*. [18] showed that white rice (pure or supplemented) was not a suitable substrate for cuticle degrading enzyme production. The results of the present study demonstrate that a mixture of solid substrate (white broken rice and rice bran) supplemented with shrimp shell waste is a potential medium for upscaling conidia production and cuticle-degrading enzyme activity in *M. koreanum* isolates. Further investigations are needed to evaluate the effects of RSM-optimized fermented conidia and their role in cuticle-degrading enzyme activity during the pathogenicity process of *M. koreanum* isolates.

## Supplemental Materials

Supplementary data for this paper are available on-line only at http://jmb.or.kr.



## Figures and Tables

**Table 1:** Variable used for single-factor experimental design.

Factor/level	Initial moisture (%)	Temperature (°C)	Substrate weight (g)	Shrimp shell waste (g)	Molasses (ml)
1	32	24	50	1.5	1.5
2	36	28	100	2.5	2.5
3	40	32	150	3.5	3.5

**Table 2:** Code and level of independent process variables used for the Box-Behnken Design optimization of conidia production.

Variables	Coded symbols	Level		
		-1	0	1
A: Initial moisture (%)	A	36	40.5	45
B: Temperature (oC)	B	24	28	32
C: Shrimp shell (g)	C	0.5	1.5	2.5
D: Incubation time (h)	D	96	216	336

**Table 3:** Different variables in Box-Behnken Design and response surface optimization result for conidia production.

Run	A	B	C	D	Y	
					Actual	Predicted
1	-1	0	1	0	7.27	7.30
2	0	1	0	1	8.22	8.30
3	1	-1	0	0	3.46	3.49
4	0	1	1	0	6.25	6.26
5	0	1	0	-1	4.25	3.95
6	1	0	0	-1	2.68	2.19
7	0	0	0	0	7.35	7.37
8	0	-1	0	1	7.87	7.84
9	1	0	1	0	3.33	3.36
10	0	0	1	1	7.54	7.53
11	0	0	0	0	7.32	7.37
12	-1	1	0	0	6.24	6.34
13	1	1	0	0	4.75	4.68
14	0	-1	0	-1	4.45	4.04
15	-1	0	-1	0	6.56	4.20
16	-1	-1	0	0	6.95	7.16
17	0	0	-1	1	8.37	8.60
18	0	0	1	-1	4.46	4.36
19	0	-1	1	0	7.15	7.16
20	-1	0	0	-1	4.05	4.28
21	0	0	0	0	7.59	7.37
22	0	1	-1	0	7.37	7.52
23	1	0	0	1	5.75	5.68
24	1	0	-1	0	5.15	4.80
25	0	0	-1	-1	3.48	3.63
26	0	0	0	0	7.08	7.37
27	-1	0	0	1	7.93	7.67
28	0	-1	-1	0	6.09	6.24
29	0	0	0	0	7.53	7.37

NB: The symbols in the table represent A (temperature), B (moisture level), C (shrimp shell), D (incubation time) and Y (Yield).
