# Supplementary material for: Optimization of Metarhizium koreanum MN031-Mt 46: Nutritional Supplementation to Improve Conidia and Cuticle-Degrading Enzyme Production by Solid-State Fermentation
Source: J Microbiol Biotechnol. 2025 Apr 27;35:e2412079. doi: 10.4014/jmb.2412.12079 (PMC12089957; doi:10.4014/jmb.2412.12079)
Supplement: Supplementary file 1 [file jmb-35-e2412079-supple.pdf]

J. Microbiol. Biotechnol. 2025. 35: e2412079
https://doi.org/10.4014/jmb.2412.12079

Supplementary Tables

Table S1. ANOVA for Quadratic model.

Source	Sum of Squares	df	Mean Square	F-value	p-value	
Model	79.31	14	5.67	60.62	< 0.0001	significant
A-Temperature	16.05	1	16.05	171.78	< 0.0001	
B-Moisture	0.1027	1	0.1027	1.10	0.3123	
C-Shrimp shell	0.0867	1	0.0867	0.9277	0.3518	
D-Incubation time	41.48	1	41.48	443.81	< 0.0001	
AB	1.00	1	1.00	10.70	0.0056	
AC	1.60	1	1.60	17.12	0.0010	
AD	0.1640	1	0.1640	1.76	0.2065	
BC	1.19	1	1.19	12.71	0.0031	
BD	0.0756	1	0.0756	0.8092	0.3836	
CD	0.8190	1	0.8190	8.76	0.0103	
A^2	14.39	1	14.39	153.98	< 0.0001	
B^2	0.9037	1	0.9037	9.67	0.0077	
C^2	0.9219	1	0.9219	9.86	0.0072	
D^2	4.95	1	4.95	52.93	< 0.0001	
Residual	1.31	14	0.0935			
Lack of Fit	1.15	10	0.1148	2.85	0.1621	not significant
Pure Error	0.1609	4	0.0402			
Cor Total	80.62	28				

Table S2. Fit Statistic

Std. Dev.	0.3057	R^2	0.9838
Mean	6.09	Adjusted R^2	0.9675
C.V. %	5.02	Predicted R^2	0.9149
		Adeq Precision	28.7562

Table S3. Student t-test result between optimized validated actual and predicted process variable data.

Variable	Levene's (Equality variances)	t-test for Equality of Means				Mean. diff	Std. Error diff	95% Confidence
	F	Sig.	t	df	Significance			
Conidia production	4.00	0.116	0.577	4	0.297 (0.595)	0.010	0.017	(-0.038, 0.058
